# Computer-controlled ion-selective electrode switch

**DOI:** 10.1155/S1463924691000238

**Published:** 1991

**Authors:** Rui A. S. Lapa, José L. F. C. Lima

**Affiliations:** Physical Chemistry Department Faculty of Pharmacy University of Porto Porto 4000 Portugal

## Abstract

The construction of a microcomputer-controlled electrode switch for use in potentiometric determinations is described. This can be coupled to most of the analytical equipment usually found in laboratories, to enable a setting up of automatic systems capable of performing sequential determinations with several ion-selective electrodes. The assessment of its analytical usage and behaviour are discussed.


Journal of Automatic Chemistry, Vol. 13, No. 3 (May–June 1991), pp. 119–122

### Computer-controlled ion-selective electrode switch

**Rui A. S. Lapa and José L. F. C. Lima***
*Physical Chemistry Department, Faculty of Pharmacy, University of Porto, 4000 Porto, Portugal*

*The construction of a microcomputer-controlled electrode switch for use in potentiometric determinations is described. This can be coupled to most of the analytical equipment usually found in laboratories, to enable a setting up of automatic systems capable of performing sequential determinations with several ion-selective electrodes. The assessment of its analytical usage and behaviour are discussed.*

### Introduction

For more than three decades, considerable efforts have been made to control analytical instrumentation with computers, although, initially, these were directed towards the control of more complex equipment. This situation has gradually changed with the advent of increasingly powerful and inexpensive microcomputers.

Easy access to microprocessors and associated devices has revolutionized chemical laboratories, so that today one can automatically perform many of the operations which in the past were done manually, a fact which is particularly attractive considering the number of tedious and time-consuming operations involved in an analytical laboratory. In the majority of the cases this is easily done by interfacing computers with available analytical equipment without having to resort to major modifications.

In electrochemistry, the potentiometric determinations, which are based on steady-state measurements, are those for which the use of microcomputers is perhaps the simplest and most viable for laboratories with limited means. They are limited to operations such as beginning the determinations, assessing and recording steady-state measurement, data treatment, and supplying the final result.

The most frequently used microcomputer-controlled potentiometric systems are automatic titrators, although they may also be applied to other types of measurements. This explains why the literature abounds with citations to different approaches [see 1–4]. In addition to the microcomputer, this type of assembly basically consists of a pH meter and a device for adding one or more reagents.

There are few papers describing potentiometric systems where the automatic system can transmit, receive, and compute values received by two or more electrodes either simultaneously or sequentially. Nevertheless, there are analytical situations in which the use of several electrodes is attractive such as in assessing the operating characteristics of new selective electrodes, namely calibrating electrodes with respect to the ion concentration, in the sequential determination of various species for which there are electrodes which can tolerate mutual interference, in determining the stability constants of complexes avoiding the usual techniques in which the glass pH electrode is used exclusively, in titrations which must be performed at a constant pH, or even the simultaneous measuring of more than one species to eliminate the difficulties resulting from electrode sensitivity, as recently suggested by Kowalski [5].

The automatic assemblies for potentiometric measurements which incorporate a multiplexer and which have been reported in the literature so far are either rigid [6–9], limited [6–10], complex [11, 12], expensive [6–8, 10, 12], or require equipment which is hard to obtain [6, 8, 9, 11, 13–15]. Also, only a few authors refer the usefulness of the assemblies when working with more than one selective electrode [9, 12, 13].

Some manufacturers (for example Orion, Crison and Corning) are currently marketing an electrodes switch which can be connected to ordinary pH meters, and, which, with a single measuring device, can perform the sequential determination of the potential supplied by several electrodes. This equipment, however, is manual.

Studies underway at the University of Porto are aimed at developing new sensors and designing new constructions for selective electrodes [16–20]; the research has demonstrated the disadvantages and limitations of systems with a manual selector and of automatic systems which only work with one electrode.

This paper describes the construction of an electrode switch, which is similar to the non-automatic commercial units, but where a microcomputer places the selective and reference electrodes in-line with a pH meter. This device can be attached to the majority of the automatic potentiometric systems available, in conjunction with a pH meter and digital burettes.

### Instrumentation

The assembly developed (see figure 1) consisted of a PC/XT computer (Super-16 Hyundai) and an RS-232C serial port which transmits and receives data, in this case to the electrode switch developed by the authors. The digital burette and pH meter used were Crison models 2031 and 2002, with RS-232C serial ports.

Output devices were a cathode ray tube, an 8-bit VGA graphic interface and a Epson FX800 dot matrix printer.

* Correspondence should be to Dr Lima.

0142–0453/91 $3.00 © 1991 Taylor & Francis Ltd.

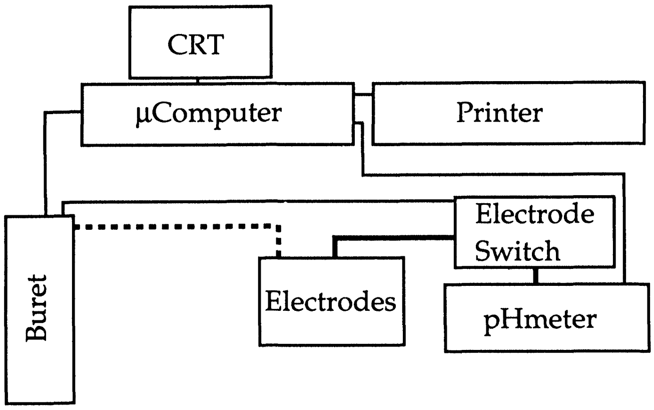

*Figure 1. Block diagram of the system.*

*The electrode switch*

As one of the essential characteristics of an electrode switch is noise immunity, it was constructed in two main blocks: one in which the serial signal is used to select different binary outputs which in turn activate the power buffer, and a second consisting of reed-relays responsible for the commutation. The entire assembly was placed in a grounded metallic box.

The first part (figure 2), which is basically required to generate the selection signal, consists of a Schimitt trigger, a rectifier, a differentiator, a counter, a latch, and of a transistor-built power drive.

The Schimitt trigger includes a NE555 integrated circuit timer to obtain a better distribution of the pulses, as they arrive to the circuit, and to increase the power of control signal. The exit signal in turn activate the counter, rectifier, and differentiator circuits.

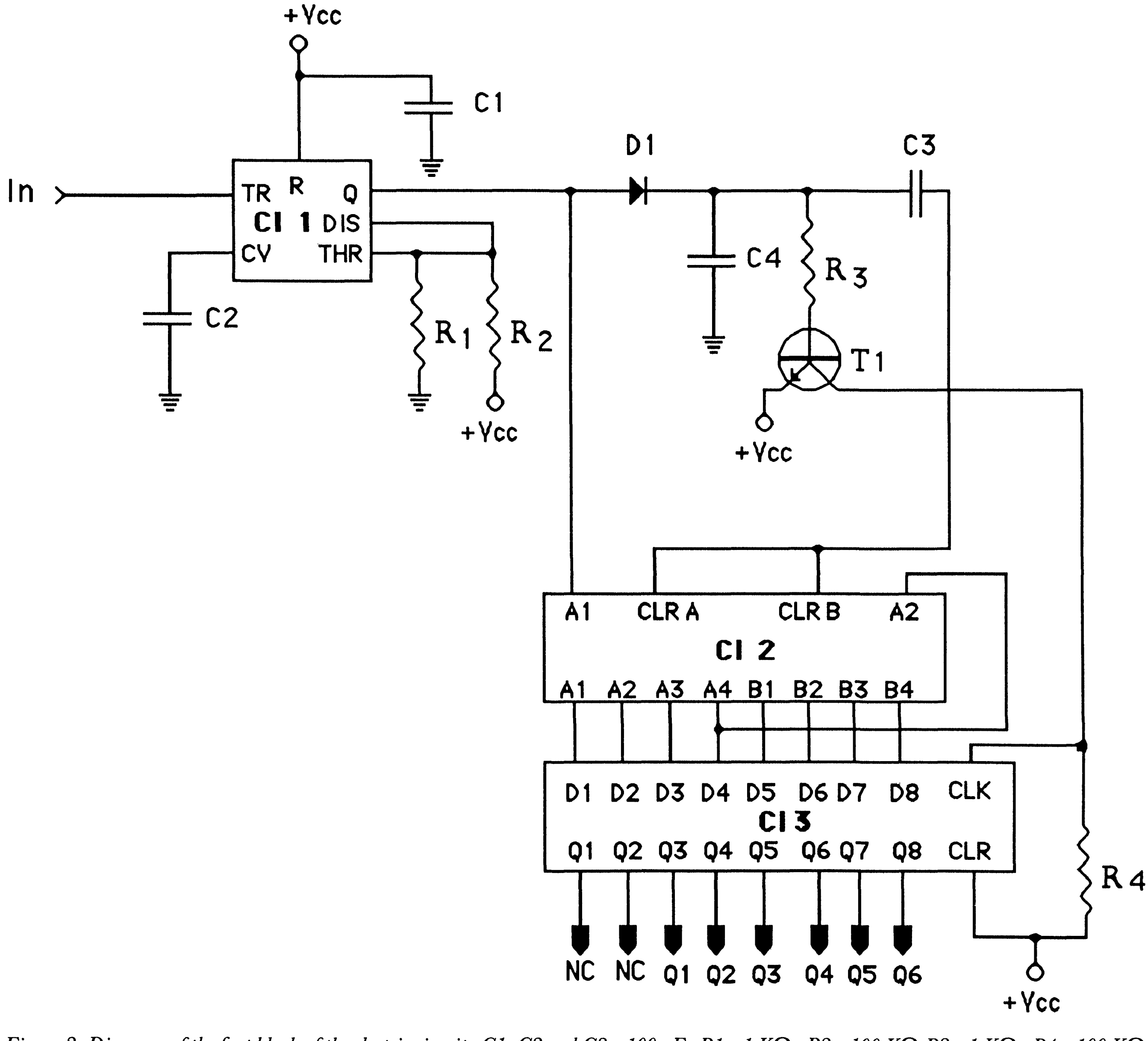

*Figure 2. Diagram of the first block of the electric circuit: C1, C2 and C3 – 100 nF; R1 – 1 KΩ; R2 – 100 KΩ R3 – 1 KΩ; R4 – 100 KΩ; T1 – BC548; D1 – 1N4146; CI 1 – NE555; CI 2 – 74LS393; CI 3 – 74LS273; + Vcc = 5 V.*

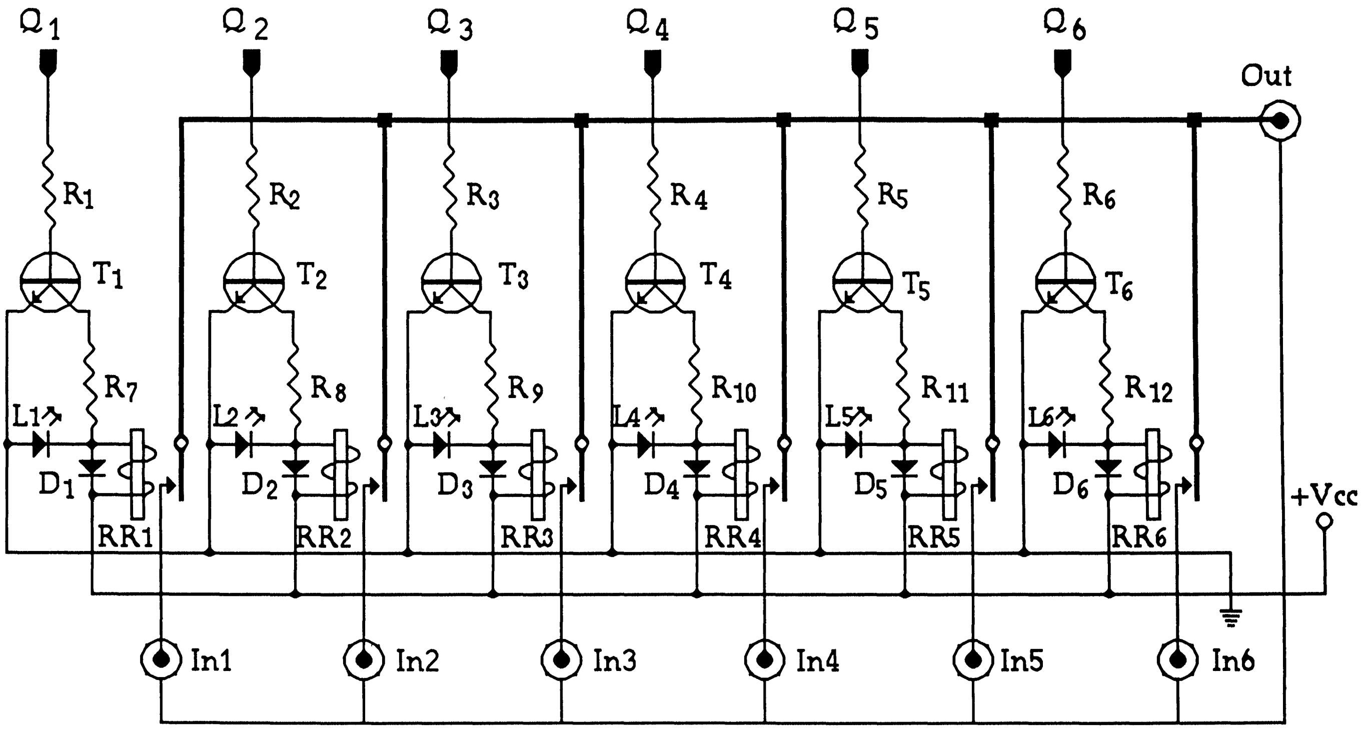

*Figure 3. Diagram of the second block of the electric circuit: R1 to R6 – 10 KΩ; R7 to R12 – 1 KΩ; L1 to L6 – LEDs; D1 to D6 – 1N4146; T1 to T6 – BC548; RR1 to RR6 – reed-relays; + Vcc = 7·5 V; Out and In1 to In6 – BNC connectors.*

The differentiator signal resets the counter when a group of pulses is sent to the line of communication; the integrator signal is responsible for activating the latch, which happens at the moment that this signal stops.

The counter circuit used was a 4+4 bit 74LS393 (TTL) double counter, which was assembled in order to attain a maximum count on the 8th bit.

The latch was mounted on the parallel output of the counter and made with a 74LS272 (TTL) (8 bits, flip-flop, type d).

The second part of the switch (figure 3) consists of six transistorized buffers ($Q_2$ to $Q_7$ of the latch) which are used to activate each of the reed relays responsible for commuting each channel. The reed relay connection was built of shielded cable.

### Control

The control programs were written in BASIC with QuickBasic (version 4.5). The sequence of instructions for controlling the electrode switch were of the type:

```

CHANNEL$(1)=MID$(STRING$(Z1,"@"),2)

CHANNEL$(2)=MID$(STRING$(Z2,"@"),2)

CHANNEL$(3)=MID$(STRING$(Z3,"@"),2)

CHANNEL$(4)=MID$(STRING$(Z4,"@"),2)

CHANNEL$(5)=MID$(STRING$(Z5,"@"),2)

CHANNEL$(6)=MID$(STRING$(Z6,"@"),2)

....

OPEN "COMX:BBBB,P,NB,ST,ASC" FOR

OUTPUT AS #N

PRINT #N,CHANNEL$(C).

```

### Discussion

The behaviour of the electrode switch was assessed by determining the more important operating characteristics with a view to the objectives for which it was designed. Switching time was less than 0·1 s, the potential differences between the various channels (all with $V_{in}$ = 0 V) were less than 5 μV, and the output potential stability (where $V_{in}$ = 0 V) was less than 1 μV/min).

This device is easily assembled from available materials and it costs, at current Portuguese market prices, approximately $20 (US), which is considerably less than other systems designed for the same purpose and mentioned earlier [6–8, 10, 12].

### Conclusions

The device described here is easily built, inexpensive, and may be used with the majority of equipment for potentiometric determination, namely those which use digital burettes and pH meters linked to a microcomputer by a RS-232C serial port.

Various electrode switches of the type described in this paper are in use in the authors' laboratory as part of assemblies used for the evaluation of selective electrodes, for potentiometric titrations, and for determining stability constants. These present definite advantages over manual systems used for the same purposes and which incorporate a commercially available, manually controlled electrode switch, a fact which has allowed the profitability and quality of laboratory work to be significantly increased.

### Acknowledgement

The authors wish to acknowledge financial support from Instituto Nacional de Investigação Científica (Portugal, Project 89/SAD/1).

### References

1. Valcárcel, M. and Luque de Castro, M., *Automatic Methods of Analysis*, Vol. 9 (Elsevier Science Publishers, Amsterdam, 1988).

2. Ward, C. R. and Reeves, J. H., *Microcomputer Applications in Chemistry* (Scott Foresman and Company, Illinois, 1990).

3. Marteel, A. E. and Motekaitis, R. S., *The Determination and Use of Stability Constants* (VCH Publishers, New York, 1988).

4. Betteridge, D. and Goad, T. B., *The Analyst,* **106** (1981), 1260.

5. Beebe, K., Uerz, D., Sandifer, J. and Kowalski, B., *Analytical Chemistry,* **46** (1974), 2111.

6. Zipper, J. J., Flee, B. and Perone, S. P., *Analytical Chemistry,* **60** (1988), 66.

7. Tan, K. H. and Reed, H. L., *Laboratory Practice,* **37** (1988), 71.

8. Gaaremstroom, P. D., English, J. C. and Perone, S. P., *Analytical Chemistry,* **50** (1978), 811.

9. Migneault, D. and Forcé, R. K., *Journal of Automatic Chemistry,* **9** (1987), 125.

10. Sandifer, J. R., Beebe, K. R. and Uerz, D. S., *Bulletin of Electrochemistry,* **4** (1988), 1061.

11. Ben-Yaakov, S., Raviv, R., Guterman, H. and Dayan, A., *Talanta,* **29** (1982), 267.

12. Reis, B. F., Godinho, O. E. S., Costa, W. F. and Aleixo, L. M., *Química Nova,* **10** (1987), 266.

13. Martin, C. R. and Freiser, H., *Analytical Chemistry,* **51** (1979), 803.

14. Wall, P., Belinger, E. G. and Brown, T., *Laboratory Practice,* **32** (1983), 88.

15. Wilmark, G., *Analytical Instrumentation,* **13** (1984), 145.

16. Lima, J. L. F. C. and Rocha, L. S. M., *International Journal of Environmental Analytical Chemistry,* **118** (1990), 127.

17. Lapa, R. A. S. and Lima, J. L. F. C., *Il Farmaco,* **45** (1990), 901.

18. Lima, J. L. F. C., Montenegro, M. C. B. S., Alonso, J., Bartroli, J. and Raurich, J. G., *Analytica Chimica Acta,* **234** (1990), 221.

19. Lima, J. L. F. C., Montenegro, M. C. B. S. and Silva, A. M. R., *Journal of Flow Injection Analysis,* **7** (1990), 19.

20. Lima, J. L. F. C., Montenegro, M. C. B. S. and Silva, A. M. R., *Journal of Pharmaceutical and Biomedical Analysis,* **8** (1990), 701.

